# The Fixed Functional Appliances in Class II Malocclusion: A Clinical Performance Comparison

**DOI:** 10.4317/jced.63954

**Published:** 2026-04-25

**Authors:** Remmiya Mary Varghese, Aravind Kumar Subramanian, Joji Alex

**Affiliations:** 1 Associate Professor, Department of Orthodontics and Dentofacial Orthopaedics, Saveetha Dental College &amp; Hospitals, Saveetha Institute of Medical and Technical Sciences, Saveetha University, Chennai, Tamilnadu, 600077; 2 Dean &amp; Head of the Department, Department of Orthodontics and Dentofacial Orthopaedics, Saveetha Dental College &amp; Hospitals, Saveetha Institute of Medical and Technical Sciences, Saveetha University, Chennai, Tamilnadu, 600077; 3 Department of Orthodontics and Dentofacial Orthopaedics, Saveetha Dental College &amp; Hospitals, Saveetha Institute of Medical and Technical Sciences, Saveetha University, Chennai, Tamilnadu, 600077

## Abstract

**Background:**

Class II malocclusion, which impact 20-25% of the population worldwide, stem from either inadequate development of the mandible or excessive development of the maxilla. Typically seen among young individuals, initiating treatment early can modify growth patterns and enhance results. Treatment Options include growth modification, dental camouflage, and surgery. Fixed functional appliances aid in better treatment outcome without relying on patient compliance. The aim of current study is to evaluate the efficiency of Forsus Fatigue Resistant Device, Powerscope, and Twin Force Bite Corrector in treating class II malocclusion.

**Material and Methods:**

A prospective study at Saveetha Dental College (Jan 2022-Jan 2023) examined 60 patients (ages 14-16) with skeletal class II malocclusion. Divided into three groups, participants received treatment with Forsus Fatigue Resistant Device, Powerscope, or Twin Force Bite Corrector. Outcomes were assessed via lateral cephalograms before and after (6-9 months) treatment.A prospective study conducted at Saveetha Dental College (Jan 2022-Jan 2023) examined 60 patients (ages 14-16) with skeletal class II malocclusion. Participants were divided into three groups and treated with either the Twin Force Bite Corrector, Powerscope, or the Forsus Fatigue Resistant Device. Lateral cephalograms were used to evaluate the outcome both before and after the treatment (6-9 months).

**Results:**

Skeletal parameter changes such as SNB, ANB, Go-Gn, Jaraback ratio, and IMPA, were significant across groups (p&lt;0.05), with TFBC showing the most efficacy in ANB (-4.69mm) change. Linear parameters like LAFH and PFH also showed significant changes, particularly in groups I and III. In dental parameters, significant changes were observed in angular measures like L1-SN, L1-NB, and interincisal angles, and linear measures such as L1-NB linear and L6-NB linear.

**Conclusions:**

Comparing the favourable outcome of the parameters TFBC is effective among the other treatment but the change in SNA and maxillary length implies a continuous and consistent follow-up and evaluation is required clinically for a favourable outcome.

## Introduction

Class II malocclusions frequently indicate an imbalance or lack of harmony between the maxilla and mandible. This typically manifests as underdevelopment of the mandible and/or overdevelopment of the maxilla, resulting in a convex soft tissue profile (1). These individuals commonly exhibit a distinctive mandibular retrognathism caused by a shortened mandible and maxillary protrusion (2). Dental and skeletal Class II malocclusion is associated with various detrimental effects, including an increased risk of dental trauma, negative perceptions of facial and dental aesthetics, decreased quality of life and self-esteem, heightened susceptibility to periodontal diseases and tooth wear, as well as a reduction in oropharyngeal space and a higher incidence of sleep disorders (3).Treating Class II malocclusion during growth stages, such as in the mixed or early permanent dentition phase, offers advantages such as the potential to modify the patient's growth pattern and decrease the likelihood of trauma to maxillary incisors. Additionally, early intervention can enhance airway space in the oropharyngeal region and achieve an optimal and stable occlusion (4). Class II malocclusion, a prevalent orthodontic issue affecting 20-25% of the global population, manifests with diverse skeletal and dental configurations across three dimensions. However, the most consistent diagnostic feature of Class II malocclusion is mandibular skeletal retrusion. Correcting Class II malocclusion involves simultaneous adjustments in the vertical and horizontal dimensions (5). Three treatment options are available for skeletal Class II malocclusion: Growth modification, Dental camouflage, and Orthognathic surgery. However, in adults growth modification cannot be carried over. Functional appliances are growth modifiers that comprise a range of orthodontic devices crafted to induce muscular forces by modifying the sagittal and vertical position of the mandible, leading to orthodontic and orthopedic alterations. Forsus Fatigue Resistant Device (FFRD), Powerscope (PS) and Twin Force Bite Corrector (TFBC) are fixed functional appliances used in the treatment of class II malocclusion. Advantage of the fixed functional appliances is that it eliminates the need for patient compliance (6). Significant mandibular elongation through Functional Jaw Orthopedics (FJO) is most effective during pubertal or immediately post-pubertal stages of skeletal development. Recent suggestions propose FJO during the pubertal growth spurt followed by fixed appliances as a viable treatment for unfavorable Class II malocclusions (7). The available literature is limited on the effectiveness of different fixed functional orthodontic devices. Thus, the aim of the present study was to analyze the effectiveness of three fixed functional appliances (Forsus Fatigue Resistant Device - FFRD, Powerscope - PS and Twin Force Bite Corrector - TFBC) within a specified duration and to compare the skeletal and dental changes.

## Material and Methods

**Figure 1:**
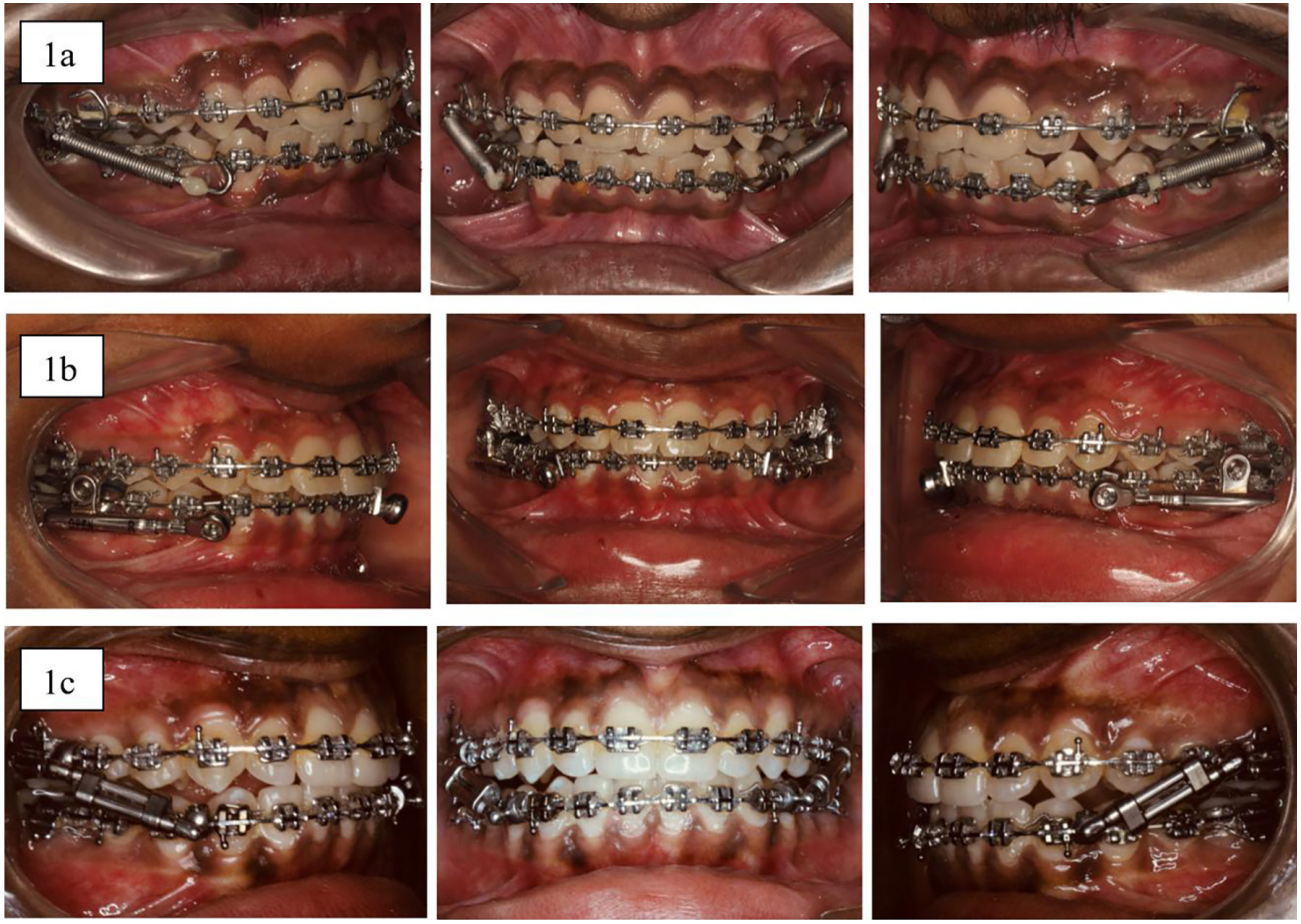
Intraoral photographs showing the three different Fixed Functional appliances; 1a – Forsus functional resistance device, 1b – Powerscope class II corrector, 1c – Twin force bite corrector.

A Prospective study was conducted at the Department of Orthodontics and Dentofacial Orthopaedics, Saveetha Dental College and Hospitals, Saveetha Institute of Medical and Technical Sciences, Saveetha University, Chennai, Tamil Nadu, India from January 2022 to January 2023. The study was reviewed and approved by the Institutional Ethical Board IHEC/SDC/FACULTY/23/ORTHO/077. Following a thorough explanation of the procedure, informed consent was obtained from all eligible patients who were willing to participate and met the inclusion criteria. Any questions or concerns raised by the patients regarding the study were addressed immediately and were clarified. The patients included in the study aged between 14 to 16 years, having skeletal class II malocclusion with Cervical Vertebral Maturation Index (CVMI) stage IV with overjet &gt;4mm, at the end of the leveling and aligning stage of a fixed multibracket orthodontic therapy. The exclusion criteria for this study includes other types of skeletal malocclusion, congenitally missing teeth, impacted teeth, malformed and retained deciduous teeth, periodontally compromised patients, medically compromised patients, previous history of active orthodontic treatment, and patients with temporomandibular disorders or any other systemic diseases affecting bone and growth. Total number of participants recruited was 72 and 12 were excluded based exclusion criteria. Remaining 60 participants were divided into three group and 20 participants were assigned in each treatment group. The treatment groups were named group I, II and III who were treated with Forsus Fatigue Resistant Device (FFRD), Powerscope (PS) and Twin Force Bite Corrector (TFBC) respectively. All the patients were treated with fixed orthodontic treatment using 0.022 MBT prescription (3M Unitek). Initial leveling and aligning was sequentially carried out up to 19 × 25 SS wire in all the patients. Before the placement of the fixed functional appliance, diagnostic records were taken after which the respective functional appliance was placed in each group. The treatment duration lasted for a duration of 6-9 months after placement of fixed functional appliance (Fig. 1).

1

Diagnostic investigations and baseline parameters such as extraoral and intraoral photographs, study models and lateral cephalograms were recorded in each group before intervention using fixed functional appliance. Lateral Cephalograms were repeated and the measurements were recorded to assess the changes after the mentioned treatment duration. The skeletal and dental study parameters included were listed in the Tables 1,2.

Table 1

Table 2

Statistical Analysis: IBM Statistical Package of Social Sciences (SPSS version 27) was used to perform the statistical analysis. One-way analysis of variance (ANOVA) is a statistical test used to assess whether there are significant differences in the means of three or more groups for a single continuous variable. A high F-statistic indicates that the intergroup variance is significantly larger than the intragroup variance, suggesting that the means of the groups are likely different. Independent t-test was used for comparisons of change in each cephalometric parameter between the treatment groups. Dental changes include correction into class 1 molar relation, proclination of the lower incisors and retroclination of the upper incisors. Skeletally the ideal changes to be expected are decrease in ANB angle, increase in mandibular length , increase in SNB angle which is an adverse effect.Mean difference between the study parameters (dental and skeletal) were considered to evaluate the effectiveness of each group.

## Results

The descriptive statistics reveal that there is a slight variation in mean age across the groups, with Group III (TFBC) having the highest mean age of 17.15 years, and Group II (PS) with a slightly lower mean age of 16.75 years (see Table 3).

Table 3

This suggests a moderate age diversity among the participants. In terms of gender distribution, there is considerable diversity among the groups. Group I (FFRD) have the highest proportion of females at 65%, while Group III (TFBC) has the lowest at 40%. Despite these variations, the overall distribution across all groups indicates a slight majority of females (53.3%) compared to males (44.7%). i) Skeletal parameters: The changes in angular cephalometric parameters such as SNB, ANB, Go-Gn, Jaraback ratio and IMPA are significantly different between the three treatment groups pre and post treatment (Table 4).

Table 4

Though the increase in SNB angle is unfavourable to the treatment outcome, the change is significant between the treatment group with group II (PS) with minimal change. Whereas the significant change in ANB is a favourable outcome with group III (TFBC) showing more efficacy. Change in linear parameters between the study groups were significantly observed in LAFH and PFH with greater change in group I and group III respectively (Table 5).

Table 5

The change in maxillary height is minimal in group III which is favourable to the treatment outcome. Comparing the skeletal angular parameters, the change in SNB angle is significant when TFBC is compared with PS and FFRD study. Similarly, the change in ANB angle is highly significant between the PS and TFBC group (p&lt;0.001). Table 5 also depicts that Go-Gn angle, jaraback ratio and IMPA angle shows significant change when group TFBC is compared with PS and FFRD that shows there is marked change in ratio between posterior and anterior facial height, mandibular plane after the treatment (Table 6).

Table 6

Though there were changes is in linear parameters pre and post treatment, none of the changes in linear parameters when compared between two groups were significant in the current study. ii) Dental parameters: The change in dental angular parameters pre and post treatment that are significant are L1-SN, L1-NB and interincisal angle (Table 7).

Table 7

All the dental linear parameters except U1-NA linear and overbite had significant change upon inter group comparison of all the three groups (Table 8).

Table 8

Comparing the dental angular parameters between two groups, the change in mean difference of L1-SN and L1-NB angle are significant between the groups FFRD vs PS and PS vs TFBC (G-I vs G-II and G-II vs G-III). The change in interincisal angle is significant in between the group II and III (see Table 9).

Table 9

Table 10 illustrates that L1-NB linear and L6-NB linear shows significant change when group II is compared with group III and group III is compared with group I.

Table 10

Also, there is significant change in overjet between group II and group III that shows there is marked change in the mandibular position (Table 10).

## Discussion

The objective of the current study is to evaluate the effectiveness of three functional appliances in a given time period. Various other studies are available that evaluate the effectiveness of functional orthodontic appliances in treating class II malocclusion, like activator and bionator (7), Forsus Fatigue Resistant Device and PowerScope (8 - 10) which has study design similar to the current study. These studies compared the pre and post treatment changes in skeletal or dental or both the parameters. Comparing the sample size of the present study, the sample size was less (N=10) in the study conducted by Kaur GJ et al. It revealed significant change in L1-NB angle between the groups which is in harmony with the present study. Whereas the same study showed significant change in IMPA angle and interincisal angle between FFRD and PS both of which is not in alignment with the current study.Another study that compared FFRD and PS demonstrated that PS had greater restraint on maxilla than FFRD which is in contrary with this study (10). Study by Arora V, et al. comparing the FFRD and PS device showed significant change in U1 and L6 linear parameters which is defiant from the current study as change in both the parameters were insignificant between the FFRD and PS group (11). Systematic review by Varghese RM et al., shows that the difference in treatment effects between FFRD and PS appliances was not significant (11). Considering the skeletal parameters, the study that shows significant change in SNA angle in Forsus device which is in contrary with the current study but the comparator treatment was functional mandibular advancer (FMA) (12). Correspondingly the study by Giuntini V, et al. showed significant restrain in SNA angle (N&gt;20). The same study had significant change in the overjet in FFRD group (-3mm) compared to twin block (13).This change is closer to the FFRD group in current study (-3.95mm) even though TFBC had greater change in overjet (-4.88mm). Similar to the current study the linear skeletal parameters, mandibular length and ramal height had no change in FFRD group when compared with the control group (14).Both the studies are showing changes in several cephalometric parameters (SNA, overjet, mandibular length and ramal height) which is in line with the changes in FFRD group in current study (15).Notably, the Twin Force Bite Corrector demonstrated the most pronounced efficacy with a substantial ANB change of -4.69mm, suggesting superior skeletal correction compared to the other devices studied.Furthermore, linear parameters such as lower anterior facial height (LAFH) and posterior facial height (PFH) exhibited significant changes, particularly evident in groups I and III. These findings highlight not only the devices' ability to impact major skeletal dimensions but also their varied effects on specific facial height measurements, crucial in assessing treatment outcomes for Class II malocclusion. Similarly, there are studies that evaluate the effectiveness of powerscope device in treating class II malocclusion (16) and changes in soft tissue, airway dimension and hyoid bone position in class II patients when treated by PS (17). Shendy M et al. study on PS had significant change in outcome parameters SNA, SNB and ANB the means of which are lesser than the current study. The change in ramal height (1.10mm) is similar to the present study (1.32mm) while other dental linear parameters have significant change but the mean is not consistent with the current study (17). In terms of dental parameters, angular measures such as L1-SN, L1-NB, and interincisal angles, along with linear measures like L1-NB linear and L6-NB linear, showed significant alterations post-treatment. These changes emphasize the devices' role in not only correcting skeletal discrepancies but also influencing dental positioning, crucial for achieving functional occlusion and aesthetic improvements. Contrary to the present study, the values of mean change in SNA, SNB and FA are greater in the study by El-Hossainy H, et al. Also, the change in other dental parameters such as LAFH, IMPA, and U1-SN are greater when compared to the PS group in the current study (18). Similar to Powerscope, there are prospective studies examining the effects of treating Class II malocclusion with the Twin Force Bite Corrector or comparing it with other functional appliances. One of the studies demonstrated significant variations in the ANB and IMPA angles when treated TFBC compared to the untreated control group, and both overjet and overbite experienced significant changes as well (19). A retrospective study revealed a noteworthy difference in the SNB angle between the FFRD and TFBC groups, consistent with our current findings. However, unlike our study, the previous one indicated a significant alteration in overbite between the FFRD and TFBC groups, which contrasts with our results. (19) In another retrospective study comparing the Twin Force Bite Corrector (TFBC) with the Jasper Jumper device, significant differences were observed. The study found a mean change in the SNB angle of 1.39° for TFBC, which is lower than the current study's finding of 2.69°. Additionally, there was a significant overjet change of -3.80mm in the TFBC group, contrasting with the current study's result of -4.88mm. The retrospective study also indicated a significant overbite change of -2.93mm in the TFBC group, whereas although overbite change wasn't significant in the present study, the mean change was -1.37mm (20). The existing literature differs from the current study, which compares three functional devices. While there are comparable cephalometric parameters for evaluating effectiveness, previous studies have limitations such as smaller sample sizes, use of different types of functional appliances for treatment, and retrospective study designs. Given the significant differences observed in treatment outcomes among the three functional devices, clinicians should consider tailoring treatment plans based on patient-specific factors such as age, gender, and craniofacial characteristics. Future research could delve deeper into understanding the variations in treatment outcomes among different functional orthodontic appliances, focusing on both skeletal and dental parameters. Current study has smaller sample size and could involve larger sample sizes to better capture the efficacy of the treatment and a longer follow-up period to evaluate the stability of the treatment. Comparing the favourable outcome of the parameters TFBC is effective among the other treatment but the change in SNA and maxillary length implies a continuous and consistent follow-up and evaluation is required clinically for a favourable outcome.Overall, the study provides comprehensive insights into the clinical efficacy of different Class II correctors, highlighting their distinct impacts on both skeletal and dental parameters. These findings contribute significantly to understanding treatment modalities for skeletal Class II malocclusion, aiding orthodontists in selecting appropriate devices based on patient-specific needs and desired treatment outcomes.

## Conclusions

At the end of the given study period meaningful clinical improvement was noted in patients with class II malocclusion who were treated with TFBC. They showed significantly higher mean change in the angular cephalometric parameters as opposed those who were treated with FFRD and PS devices. The linear parameters also improved (noteworthy changes in LAFH and PFH) in those treated with TFBC and FFRD compared to PS. The choice between PowerScope Class II Corrector, Forsus Fatigue Resistant Device, and Twin Force Bite Corrector depends on various factors including patient age, compliance, severity of malocclusion, and clinician preference. Each appliance has its strengths in clinical efficiency, and the decision should be made in consultation with an experienced orthodontist who can tailor the treatment plan to achieve optimal outcomes for the patient's specific condition.

## Figures and Tables

**Table 1:** Skeletal parameters for treatment efficiency analysis.

Skeletal Parameters		Description
Angular parameters	SNA	Angle formed by joining the sella, nasion, and A point. It evaluates the anteroposterior position of the maxilla to the anterior cranial base
	SNB	Angle is formed by joining the sella to nasion to B point. It evaluates the anteroposterior position of the mandible to the anterior cranial base
	ANB	Difference between SNA and SNB. It measures the anteroposterior relationship between the maxilla and the mandible.
	Go-Gn	Angle formed between the goniongnathion plane and the sellanasion plane
	Jaraback ratio	Ratio of posterior facial height to anterior facial height
	IMPA	Inner angle formed between the long axis of mandibular central incisor and the mandibular plane
	FA	The angle formed by the facial axis (Pt-Gn) and the Basion - Nasion plane
Linear Parameters	UAFH	Upper anterior facial height is the direct measurement from the nasion to the gonion along the true vertical plane
	LAFH	Lower anterior facial height is the direct measurement from gonion to menton along the true vertical plane
	PFH	Posterior facial height is the measure from sella to gonion
	TAFH	Anterior facial height is the measure from nasion to menton
	Ramal Height	It is measured from the superior most part of condyle to the lower border of the mandible.
	Maxillary Length	It is linear distance measured between anterior and posterior nasal spine
	Mandibular Length	It is the linear distance measured from the Co- Go

1

**Table 2:** Dental parameters for treatment efficiency analysis.

Dental Parameters		Description
Angular parameters	U1- SN	Angle between the long axis of upper incisor and the SN plane
	U1-NA	Angle between the long axis of upper incisor and the N-B line
	L1-SN	Angle between the long axis of upper incisor and the SN plane
	L1-NB	Angle between the long axis of lower incisor and the N-B line
	Interincisal angle	Angle formed by long axis of the upper incisor and long axis of lower incisor
Linear Parameters	U1 NA linear	It is the perpendicular distance measured from the incisal edge of the uppercentral incisor to the N-A line
	U6-NA linear	It is the perpendicular linear measurement taken from the mesiobuccal cusp of the upper first molar to the N-A line
	L1-NB linear	It is the perpendicular distance measured from the incisal edge of the lower central incisor to the N-B line
	L6-NB linear	It is the perpendicular linear measurement taken from the mesiobuccal cusp of the lower first molar to the N-B line
	Overjet	It is measured as the distance between the upper central incisor and the lower incisor in the horizontal plane
	Overbite	It is measured as the distance between the upper central incisor and the lower incisor in the vertical plane

2

**Table 3:** Age and gender distribution of study participants.

Group	Age (years)				Gender	
	N	Mean	Std. Deviation	Std. Error	Female (N, %)	Male (N, %)
Group -I (FFRD)	20	17.00	2.05	0.46	13 (65%)	7 (35%)
Group-II (PS)	20	16.75	2.29	0.51	11 (55%)	9 (45%)
Group-III (TFBC)	20	17.15	2.32	0.52	8 (40%)	12 (60%)
Total	60	16.97	2.19	0.28	32 (53.3%)	28 (44.7%)

3

**Table 4:** One way ANOVA to compare change in skeletal angular parameters within the groups.

Angular parameters	Study Group	N	Mean change (degree)	Std. Deviation	Std. Error	ANOVA	
						F	p-value
SNA	(G-I) FFRD	20	-2.09	3.06	0.68	0.62	0.542
	(G-II) PS	20	-1.19	1.54	0.34		
	(G-III) TBC	20	-2.02	3.54	0.79		
SNB	(G-I) FFRD	20	0.89	1.79	0.4	9.653	<0.001**
	(G-II) PS	20	-0.01	1.06	0.24		
	(G-III) TFBC	20	2.69	2.72	0.61		
ANB	(G-I) FFRD	20	-2.73	3.06	0.69	8.923	<0.001**
	(G-II) PS	20	-1.15	1.9	0.42		
	(G-III) TFBC	20	-4.69	2.86	0.64		
Go-Gn	(G-I) FFRD	20	0.58	1.82	0.41	24.306	<0.001**
	(G-II) PS	20	-0.72	1.74	0.39		
	(G-III) TFBC	20	-5.25	4.1	0.92		
Jaraback ratio	(G-I) FFRD	20	-1.5	1.93	0.43	28.536	<0.001**
	(G-II) PS	20	-1.15	2.64	0.59		
	(G-III) TFBC	20	3.58	2.49	0.56		
IMPA	(G-I) FFRD	20	5.25	3.79	0.85	13.439	<0.001**
	(G-II) PS	20	2.07	5.04	1.13		
	(G-III) TFBC	20	9.94	5.5	1.23		
FA	(G-I) FFRD	20	-1.06	2.23	0.5	0.341	0.713
	(G-II) PS	20	-1.11	4.62	1.03		
	(G-III) TFBC	20	-0.49	2.59	0.58		

*p value <0.05- significant; **p value <0.001- highly significant

**Table 5:** One way ANOVA to compare change in skeletal linear parameters within the groups.

Linear Parameters	Study Group	N	Mean change (mm)	Std. Deviation	Std. Error	ANOVA	
						F	p-value
UAFH	(G-I) FFRD	20	3.5	9.65	2.16	1.328	0.273
	(G-II) PS	20	-0.19	7.76	1.73		
	(G-III) TFBC	20	1.22	1.84	0.41		
LAFH	(G-I) FFRD	20	2.76	9.87	2.21	4.348	0.017*
	(G-II) PS	20	-1.99	6.41	1.43		
	(G-III) TFBC	20	1.36	5.29	1.18		
PFH	(G-I) FFRD	20	3.72	14.03	3.14	3.751	0.029*
	(G-II) PS	20	-0.54	3.69	0.83		
	(G-III) TFBC	20	4.18	3.85	0.86		
TAFH	(G-I) FFRD	20	-1.67	7.22	1.61	2.846	0.066
	(G-II) PS	20	-0.37	4.95	1.11		
	(G-III) TFBC	20	2.83	6.04	1.35		
Ramal Height	(G-I) FFRD	20	0.55	7.07	1.58	1.933	0.154
	(G-II) PS	20	1.32	2.71	0.61		
	(G-III) TFBC	20	3.58	4.43	0.99		
Maxillary Length	(G-I) FFRD	20	-1.81	9.25	2.07	2.787	0.07
	(G-II) PS	20	3.18	4.39	0.98		
	(G-III) TFBC	20	0.73	5.41	1.21		
Mandibular Length	(G-I) FFRD	20	-0.84	9.37	2.1	1.682	0.195
	(G-II) PS	20	3.03	6.11	1.37		
	(G-III) TFBC	20	2.15	4.65	1.04		

*p value <0.05- significant; **p value <0.001- highly significant

**Table 6:** T-test to compare significant change in skeletal angular parameters between the groups.

Angular parameter	Comparator group		Mean Difference	Std. Error	P- value	

SNA	G-I vs G-II	FFRD vs PS	0.90	0.90	0.58	
	G-II vs G-III	PS vs TFBC	0.83	0.90	0.63	
	G-III vs G-I	TFBC vs FFRD	0.07	0.90	0.997	
SNB	G-I vs G-II	FFRD vs PS	-0.90	0.63	0.33	
	G-II vs G-III	PS vs TFBC	-2.70	0.63	<0.001**	
	G-III vs G-I	TFBC vs FFRD	1.80	0.63	0.015*	
ANB	G-I vs G-II	FFRD vs PS	1.58	0.84	0.15	
	G-II vs G-III	PS vs TFBC	3.54	0.84	<0.001**	
	G-III vs G-I	TFBC vs FFRD	-1.96	0.84	0.059	
Go-Gn	G-I vs G-II	FFRD vs PS	-1.30	0.88	0.31	
	G-II vs G-III	PS vs TFBC	4.53	0.88	0.001*	
	G-III vs G-I	TFBC vs FFRD	-5.83	0.88	<0.001**	
Jaraback ratio	G-I vs G-II	FFRD vs PS	0.35	0.75	0.89	
	G-II vs G-III	PS vs TFBC	-4.73	0.75	<0.001**	
	G-III vs G-I	TFBC vs FFRD	5.08	0.75	<0.001**	
IMPA	G-I vs G-II	FFRD vs PS	-3.18	1.53	0.10	
	G-II vs G-III	PS vs TFBC	-7.87	1.53	<0.001**	
	G-III vs G-I	TFBC vs FFRD	4.69	1.53	<0.009*	
FA	G-I vs G-II	FFRD vs PS	-0.05	1.05	1.00	
	G-II vs G-III	PS vs TFBC	-0.62	1.05	0.83	
	G-III vs G-I	TFBC vs FFRD	0.57	1.05	0.850	

6

**Table 7:** One way ANOVA to compare change in dental angular parameters within the groups.

Angular parameter	Study Group	N	Mean (degree)	Std. Deviation	Std. Error	ANOVA	
						F	p-value
U1- SN	(G-I) FFRD	20	0.56	1.7	0.38	0.27	0.765
	(G-II) PS	20	0.23	1.47	0.33		
	(G-III) TBC	20	0.27	1.47	0.33		
U1-NA	(G-I) FFRD	20	-1.91	4.68	1.05	0.276	0.76
	(G-II) PS	20	-1.77	3.89	0.87		
	(G-III) TFBC	20	-0.67	7.96	1.78		
L1-SN	(G-I) FFRD	20	-0.9	1.49	0.33	7.506	0.001**
	(G-II) PS	20	0.41	1.35	0.3		
	(G-III) TFBC	20	-1.2	1.35	0.3		
L1-NB	(G-I) FFRD	20	6.15	3.78	0.85	13.007	<0.001**
	(G-II) PS	20	1.6	4.85	1.08		
	(G-III) TFBC	20	8.92	5.02	1.12		
Interincisal angle	(G-I) FFRD	20	-1.07	4.69	1.05	3.711	0.031*
	(G-II) PS	20	1.56	5.65	1.26		
	(G-III) TFBC	20	-3.62	7.38	1.65		

*p value <0.05- significant; **p value <0.001- highly significant

**Table 8:** One way ANOVA to compare change in dental linear parameters within the groups.

Linear parameter	Study Group	N	Mean (mm)	Std. Deviation	Std. Error	ANOVA	
						F	p-value
U1 NA linear	(G-I) FFRD	20	1.34	3.67	0.82	2.855	0.066
	(G-II) PS	20	-0.59	1.83	0.41		
	(G-III) TBC	20	-1.14	4.34	0.97		
U6-NA linear	(G-I) FFRD	20	-0.65	4.96	1.11	13.771	<0.001**
	(G-II) PS	20	-1.41	3.52	0.79		
	(G-III) TFBC	20	0.75	1.09	0.24		
L1-NB linear	(G-I) FFRD	20	1.37	1.55	0.35	12.439	<0.001**
	(G-II) PS	20	0.09	1.55	0.35		
	(G-III) TFBC	20	-1.43	2.17	0.48		
L6-NB linear	(G-I) FFRD	20	-0.3	5.63	1.26	9.188	<0.001**
	(G-II) PS	20	-1.9	4.28	0.96		
	(G-III) TFBC	20	-8.97	9.43	2.11		
Overjet	(G-I) FFRD	20	-3.95	3.2	0.71	4.104	0.022*
	(G-II) PS	20	-2.49	1.64	0.37		
	(G-III) TFBC	20	-4.88	2.89	0.65		
overbite	(G-I) FFRD	20	-1.09	1.01	0.23	0.673	0.514
	(G-II) PS	20	-1.68	1.64	0.37		
	(G-III) TFBC	20	-1.37	2.01	0.45		

*p value <0.05- significant; **p value <0.001- highly significant

**Table 9:** T-test to compare significant change in dental angular parameters between the groups.

Angular parameter	Comparator group		Mean Difference	Std. Error	P- value
U1- SN	G-I vs G-II	FFRD vs PS	0.33	0.49	0.780
	G-II vs G-III	PS vs TFBC	-0.04	0.49	0.996
	G-III vs G-I	TFBC vs FFRD	-0.29	0.49	0.825
U1-NA	G-I vs G-II	FFRD vs PS	-0.14	1.83	0.997
	G-II vs G-III	PS vs TFBC	-1.10	1.83	0.820
	G-III vs G-I	TFBC vs FFRD	1.24	1.83	0.777
L1-SN	G-I vs G-II	FFRD vs PS	-1.31	0.44	0.012*
	G-II vs G-III	PS vs TFBC	1.61	0.44	0.002*
	G-III vs G-I	TFBC vs FFRD	-0.30	0.44	0.777
L1-NB	G-I vs G-II	FFRD vs PS	4.55	1.45	0.007*
	G-II vs G-III	PS vs TFBC	-7.32	1.45	<0.001**
	G-III vs G-I	TFBC vs FFRD	2.77	1.45	0.145
Interincisal angle	G-I vs G-II	FFRD vs PS	-2.63	1.90	0.356
	G-II vs G-III	PS vs TFBC	5.18	1.90	0.023*
	G-III vs G-I	TFBC vs FFRD	-2.55	1.90	0.379

*p value <0.05- significant; **p value <0.001- highly significant

**Table 10:** T-test to compare significant change in dental linear parameters between the groups.

Dependent Variable	Comparator group		Mean Difference	Std. Error	P- value	

U1 NA linear	G-I vs G-II	FFRD vs PS	1.93	1.09	0.189	
	G-II vs G-III	PS vs TFBC	0.55	1.09	0.869	
	G-III vs G-I	TFBC vs FFRD	-2.48	1.09	0.068	
U6-NA linear	G-I vs G-II	FFRD vs PS	0.76	1.13	0.782	
	G-II vs G-III	PS vs TFBC	-2.16	1.13	0.145	
	G-III vs G-I	TFBC vs FFRD	1.40	1.13	0.434	
L1-NB linear	G-I vs G-II	FFRD vs PS	1.28	0.56	0.067	
	G-II vs G-III	PS vs TFBC	1.52	0.56	0.024*	
	G-III vs G-I	TFBC vs FFRD	-2.80	0.56	<0.001**	
L6-NB linear	G-I vs G-II	FFRD vs PS	1.60	2.15	0.739	
	G-II vs G-III	PS vs TFBC	7.07	2.15	0.005*	
	G-III vs G-I	TFBC vs FFRD	-8.67	2.15	<0.001**	
Overjet	G-I vs G-II	FFRD vs PS	-1.46	0.84	0.201	
	G-II vs G-III	PS vs TFBC	2.39	0.84	0.017*	
	G-III vs G-I	TFBC vs FFRD	-0.93	0.84	0.514	
Overbite	G-I vs G-II	FFRD vs PS	0.59	0.51	0.482	
	G-II vs G-III	PS vs TFBC	-0.31	0.51	0.816	
	G-III vs G-I	TFBC vs FFRD	-0.28	0.51	0.847	

*p value <0.05- significant; **p value <0.001- highly significant
